# Case Report: Blurring the immunological border in the diagnostic evaluation of optic neuritis

**DOI:** 10.3389/fimmu.2026.1824655

**Published:** 2026-10-22

**Authors:** Elisa Basili, Valentina Annamaria Mauceri, Alessandro Di Paola, Marco Zoccarato, Francesca Rinaldi, Paola Perini, Chiara Briani, Marco Puthenparampil

**Affiliations:** 1 Department of Neurosciences, University of Padua, Padua, Italy; 2 Neuroradiology Unit, University Hospital of Padua, Padua, Italy; 3 Multiple Sclerosis Centre, Day Hospital and Centre for Advanced Neurological Therapies Unit, University Hospital of Padua, Padua, Italy

**Keywords:** antiphospholipid syndrome, MOG-associated disease, MRI, multiple sclerosis, OCT, optic neuritis, optic perineuritis

## Abstract

**Background:**

Optic neuritis (ON) is a common inflammatory syndrome that may represent the initial manifestation of multiple sclerosis (MS), myelin oligodendrocyte glycoprotein antibody–associated disease (MOGAD), or other autoimmune disorders. Although serological testing and imaging criteria have improved diagnostic accuracy, early immunological and radiological findings may overlap, creating diagnostic uncertainty with relevant therapeutic implications.

**Case presentation:**

A 48-year-old woman presented with sudden, painless monocular visual loss. Orbital MRI showed optic perineuritis with perineural dural enhancement. The combination of optic perineuritis and subsequent optic nerve involvement initially suggested a MOGAD-like phenotype or Ischemic Optic Neuropathy. Although extensive immunological screening found a strong positivity of anti-β_2_glycoprotein1 and anti-cardiolipin IgM antibodies, which prompted a rheumatological evaluation to exclude systemic autoimmune diseases like antiphospholipid syndrome (APS), fluorescein angiography was negative. Moreover, both anti-aquaporin-4 antibodies and anti- MOG antibodies (tested with also live cell-based assays) were negative. On the other hand, cerebrospinal fluid analysis revealed CSF-restricted IgG oligoclonal bands (Pattern II). High-dose intravenous methylprednisolone produced partial clinical improvement, but visual deficit persisted. During longitudinal follow-up, OCT demonstrated ganglion cell layer thinning, while a further MRI revealed a new juxtacortical lesion, fulfilling dissemination in space criteria and supporting a diagnosis of multiple sclerosis. Therefore, anti-CD20 therapy was initiated due to persistent disability.

**Discussion:**

This case illustrates how early immunological and radiological red flags may suggest other systemic autoimmune diseases despite eventual confirmation of MS. The diagnostic pathway was shaped by the temporal evolution of imaging and biomarker findings rather than by isolated baseline features. The observation emphasizes the risk of premature diagnostic labelling in seronegative optic neuritis and highlights the need for cautious interpretation of early red flags within a longitudinal framework.

**Conclusion:**

At the immunological border in the diagnostic evaluation of optic neuritis, early phenotypic presentations may not reliably predict final disease classification. Longitudinal integration of clinical evolution, imaging findings, and biomarker data is essential to achieve diagnostic certainty and guide appropriate long-term treatment decisions. This case supports a dynamic, evidence-based diagnostic approach in atypical immune-mediated optic neuritis.

## 1 Introduction

Optic neuritis (ON) is a syndrome that may characterize the onset of Multiple Sclerosis (MS) (1), anti-AQP4 Neuromyelitis Optica (NMO) (2), and MOG-Associated Disorder (MOGAD) (3). Recent diagnostic criteria have standardized the identification of clinical and paraclinical features supporting the diagnosis of optic neuritis, improving diagnostic consistency and guiding early therapeutic decisions. Among paraclinical tests, the detection of specific autoantibodies drives both the diagnosis of ON and the diagnosis of MOGAD and AQP4-NMO, while IgG Oligoclonal Bands (IgGOB) might be supportive for MS diagnosis, but not always required. Unfortunately, antibody detection requires time, while other paraclinical tests supporting ON may provide immediate results (OCT and MRI). Therefore, even if MS diagnosis is considerably more frequent, it is important to look at MRI and OCT, evaluating the presence of red flags that might increase the probability of NMO, MOGAD or other systemic autoimmune diseases.

Here we present a case of optic perineuritis (OPN), which subsequently evolved into ON, with clinical and MRI findings initially supporting the hypothesis of MOGAD or Ischemic Optic Neuropathy. Nevertheless, the immunological screening revealed persistent high-titer antiphospholipid antibodies, while fluorescein angiography was negative, as well as the tests anti-AQP4 and for anti-MOG antibodies (performed also with live-Cell Based Assay, l-CBA), questioning MOGAD or ION. After one year, the appearance of a new juxtacortical lesion, together with the longitudinal clinical and laboratory findings, allowed the final diagnosis of MS. This case illustrates how immunological and radiological red flags may precede the eventual diagnosis of multiple sclerosis, highlighting the importance of longitudinal integration of clinical, imaging, and biomarker data to obtain a definitive diagnosis.

## 2 Case description and diagnostic assessment

A 48-year-old woman presented to the Emergency Department in February 2024 for a sudden, painless, and rapidly worsening (in a few hours) monocular vision loss in the Left Eye (LE) upon awakening. She described the vision loss as a central “white cloud” with progressive enlargement to the visual field periphery. Her past medical history was significant for severe myopia in the left eye and for previous posterior vitreous detachment in the same eye, so she first underwent an ophthalmological evaluation, which did not describe any further relevant findings, except for a positive Relative Afferent Pupillary Defect (RAPD) in the left eye. Although the onset of visual loss was sudden and painless, the suspicion of acute optic neuritis arose from the presence of RAPD in the absence of retinal abnormalities.

A brain MRI was performed, showing thickening and perineural dural contrast enhancement of the left optic nerve, in the absence of any focal signal abnormalities along the optic nerve, as well as a single, temporal periventricular white matter lesion. Furthermore, Diffusion-weighted imaging (DWI) and apparent diffusion coefficient (ADC) sequences did not demonstrate diffusion restriction within this lesion, arguing against an acute ischemic lesion (**Figure 1**).

**Figure 1:**
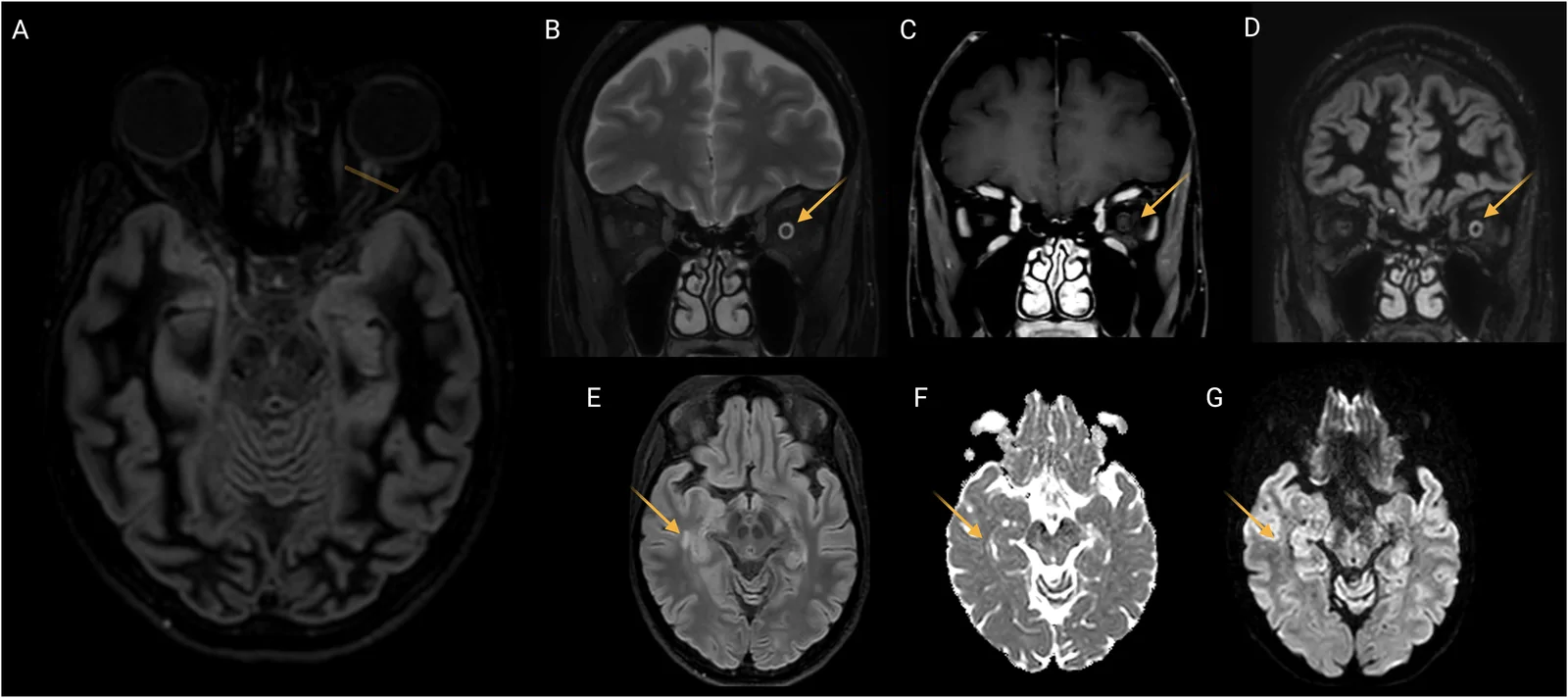
Baseline MRI showing optic perineuritis. Brain MRI performed at symptom onset. **(A)** Axial DIR sequence showing abnormal signal surrounding the left optic nerve. **(B, C)** Coronal T2-weighted and contrast-enhanced images demonstrating perineural enhancement of the left optic nerve sheath (arrow), consistent with optic perineuritis. **(D)** Coronal DIR sequence confirming signal alteration around the optic nerve. **(E)** Axial FLAIR, ADC **(F)** and DWI **(G)** sequences showing a small temporal periventricular white matter lesion (arrow), insufficient at baseline to meet dissemination in space criteria for multiple sclerosis. Susceptibility-based MRI biomarkers of multiple sclerosis, including the central vein sign and paramagnetic rim lesions, could not be reliably assessed on the available MRI sequences. Created in BioRender. Puthenparampil, (M) (2026) https://BioRender.com/7inevjs.

The diagnostic work-up was completed by Visual Evoked Potential (VEP), which showed absence of response following stimulation in the LE, and Optical Coherence Tomography (OCT), which did not show any asymmetry.

Since the MRI findings were suggestive of OPN, four days after symptom onset, and before the administration of corticosteroids or any other immunosuppressive treatment, a complete immunological screening to exclude systemic disorders (Routine blood tests, B12 vitamin, folates and Angiotensin Converting Enzyme concentrations were normal; immunological screenings including ANA, ANCA, ENA, anti-dsDNA, anti-b_2_glycoprotein1, anti-cardiolipin and LAC, anti-AQP4, and MOGAD (anti-MOG) was performed (4). Because the initial result was negative, MOG-IgG testing was repeated on previously stored sample, using a l-CBA (5). Only pathological concentrations of anti-β_2_glycoprotein1 and anti-cardiolipin IgM antibodies (264.8 KU/l, positive if >10.0, and 158.7 MPL-KU/l, positive if > 40.0 respectively) were observed. In addition, a lumbar puncture was performed, with no evidence of any red flags (cell count: 7 lymphocytes/μL; Q_ALB_ 5.3x10^-3^) but of CSF-restricted IgG Oligoclonal Bands (Pattern II). Then, retinal fluorescein angiography was performed and did not reveal any evidence of an ischemic etiology.

Although clinical and laboratorial findings supported the diagnosis of possible optic neuritis (6), the patient was then treated with high-dose intravenous Methylprednisolone. Since anti-MOG antibodies were negative, oral steroid tapering was completed in 2 weeks, with only partial visual improvement and persistent (even if reduced) central scotoma. Due to the strong positivity of anti-β_2_glycoprotein1 and anti-cardiolipin IgM antibodies, which was confirmed after 12 weeks, the patient underwent a rheumatological evaluation and then started antiplatelet therapy for primary prevention, given the absence of any previous thrombotic or obstetric events. After one month, a new brain MRI was performed and showed a reduction of perineural dural contrast enhancement of the optic nerve, together with the evidence of a hyperintense lesion (< 1cm) in the left optic nerve on DIR sequences (**Figure 2**). At this stage, the diagnosis of optic neuritis was established based on the MRI lesion and by the presence of CSF-restricted IgGOB (6). However, it was not possible to give a final diagnosis of MS since only one temporal periventricular lesion was observed and at that time the optic nerve was not counted for dissemination in space criteria (7).

**Figure 2:**
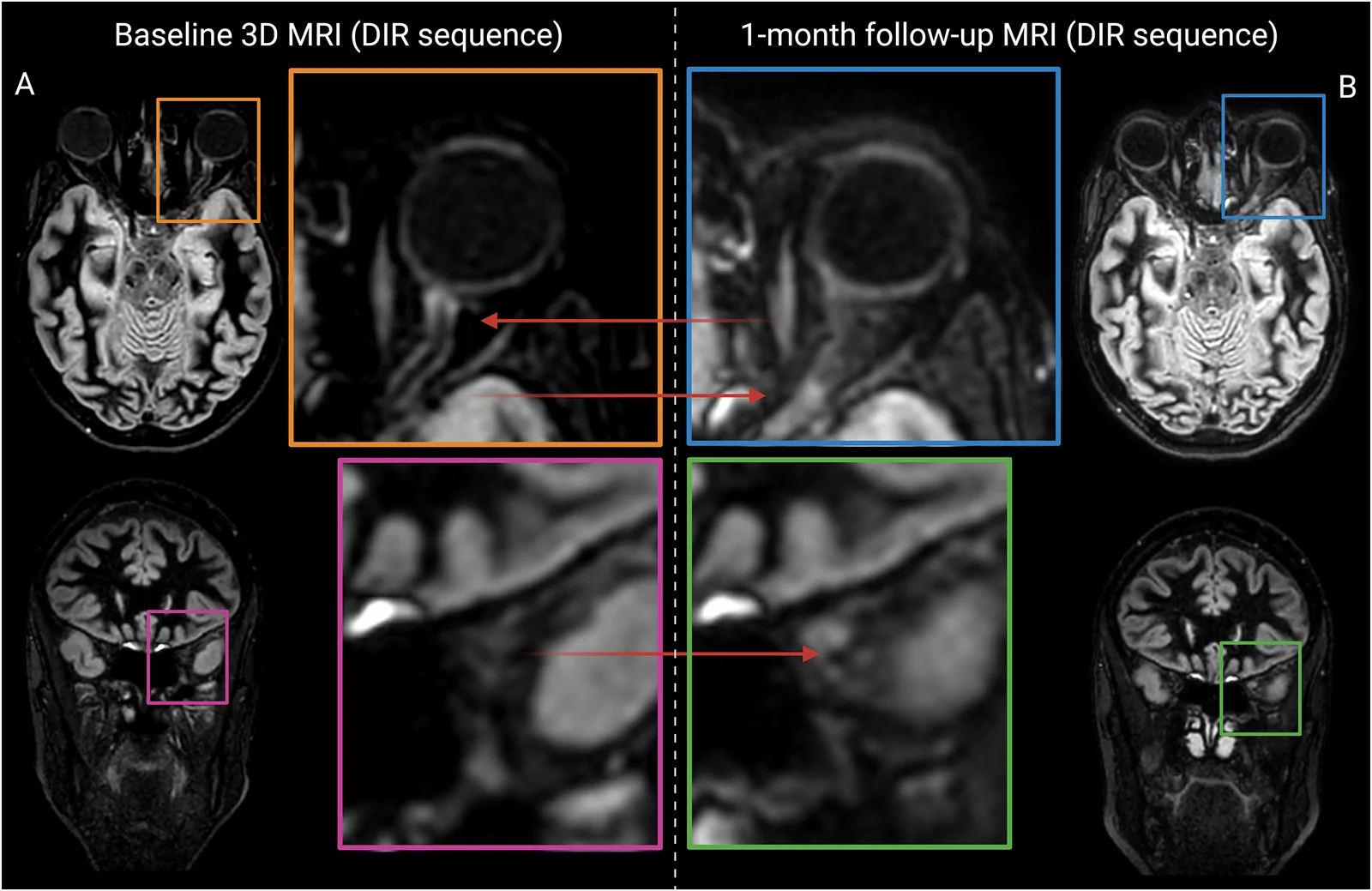
Radiological evolution from optic perineuritis to optic neuritis. Comparison between baseline MRI **(A)** and the 1-month follow-up MRI **(B)** DIR sequences. Baseline images show signal alteration surrounding the left optic nerve, consistent with optic perineuritis, with no evidence of optic nerve involvement. At follow-up, increased signal involving the optic nerve itself was observed (arrows), indicating evolution toward optic neuritis, while OPN was no longer identified. Created in BioRender. Puthenparampil, (M) (2026) https://BioRender.com/r5iu82s.

After 2 months, the patient reported the persistence of visual field defect and OCT disclosed an important reduction of both peripapillary retinal nerve fiber layer (pRNFL) thickness and macular ganglion cell layer (mGCL) and inner plexiform layer (mIPL) volumes (**Figure 3**). All these findings, together with the OPN observed at onset, were not typical of MS.

**Figure 3:**
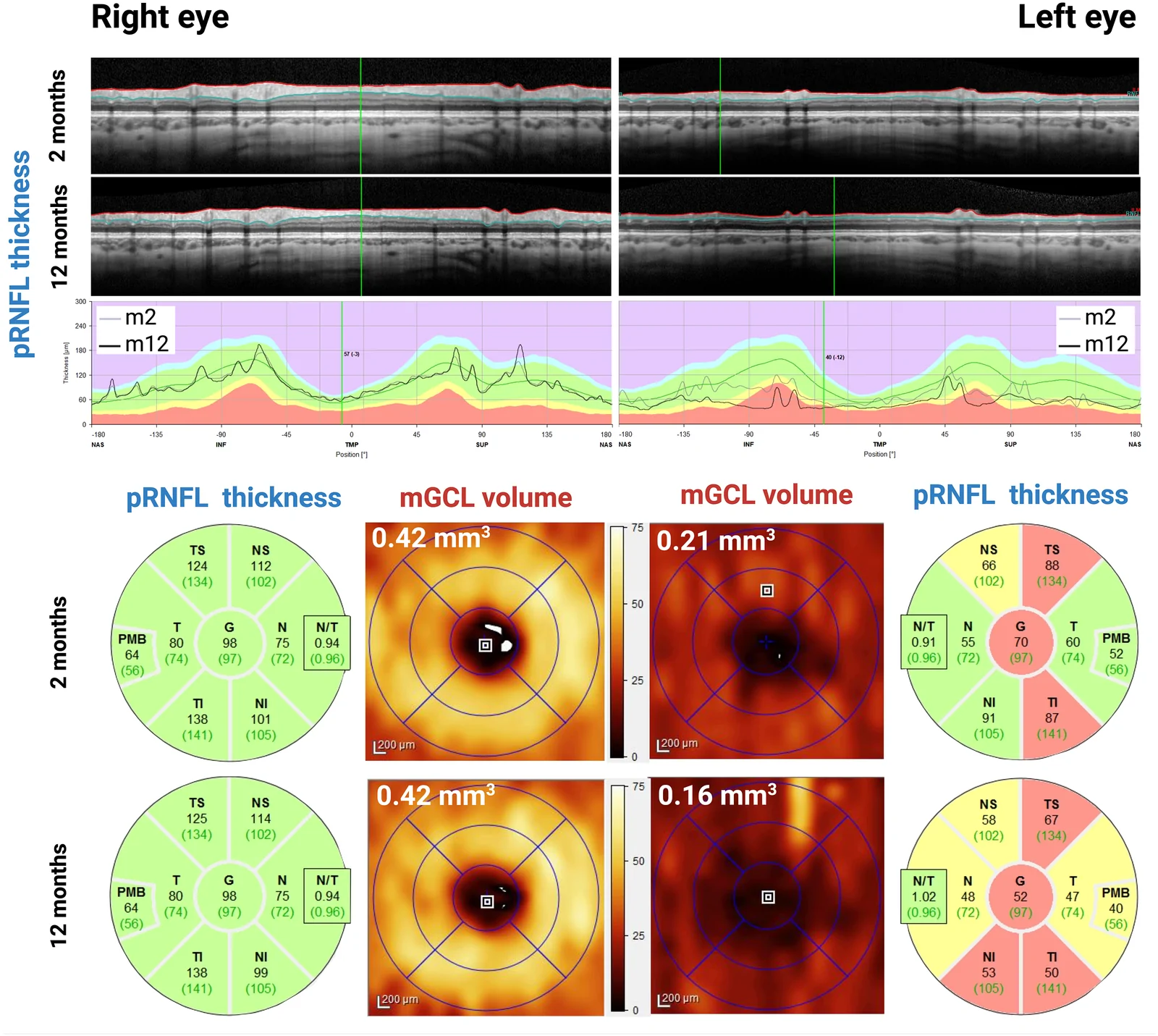
Longitudinal OCT changes after optic neuritis. Optical coherence tomography (OCT) of the right eye (unaffected) and left eye (affected) performed at 2 and 12 months after symptom onset. The right eye shows stable peripapillary retinal nerve fiber layer (pRNFL) thickness and macular ganglion cell layer (mGCL) volume. In contrast, the affected left eye demonstrates progressive pRNFL thinning and marked reduction of mGCL volume over time, consistent with axonal loss following optic neuritis. Created in BioRender. Puthenparampil, M. (2026) https://BioRender.com/hawnlks.

After 6 months, brain MRI did not show any new lesion, and thus the patient also continued rheumatological follow-up.

A further follow-up brain MRI was performed in March 2025 and confirmed diffuse signal alteration of the left optic nerve on DIR and FLAIR sequences as well as the presence of a new, right temporal juxtacortical white matter lesion (**Figure 4**). The OCT evaluation found a further reduction of pRNFL thickness and mGCL and mIPL volumes (**Figure 3**). VEPs showed again the complete absence of any wave when stimulating the left eye, while Multifocal electroretinography (mfERG) revealed delayed nerve conduction along large axons and the papillomacular bundle. Although the clinical presentation was atypical (painless optic neuritis associated with OPN) with relevant retinal thinning, the identification of a new juxtacortical lesion together with the exclusion of better explanations allowed the acquisition of dissemination in space, supporting the final diagnosis of MS. Moreover, no better explanation was supported by the absence of any anti-MOG or AQP4 antibodies (tested before treatment, anti-MOG also with l-CBA) (5) and normal fluorescein angiography.

**Figure 4:**
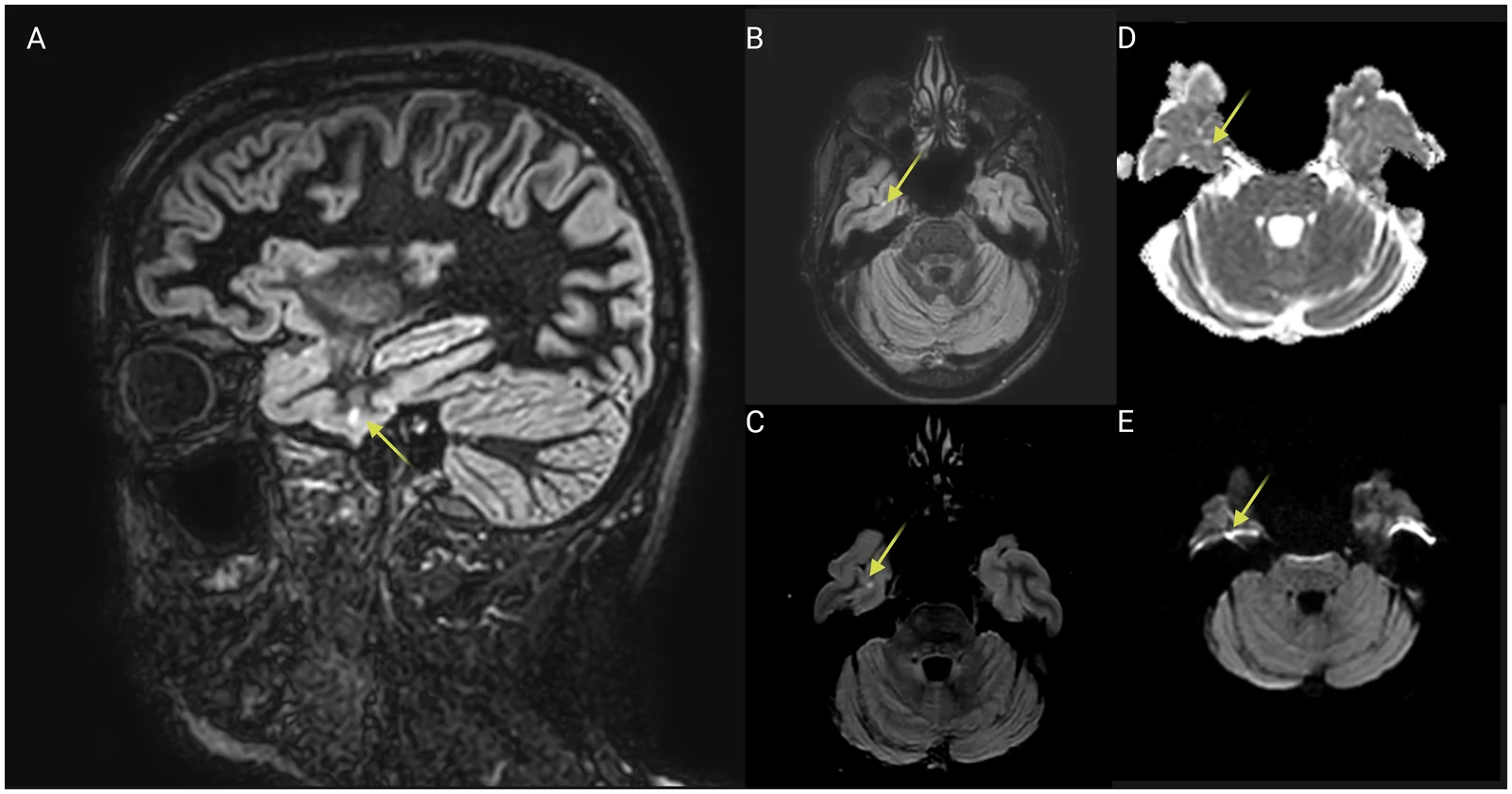
New juxtacortical lesion on follow-up brain MRI. **(A)** Sagittal DIR image showing a small right temporal juxtacortical white matter lesion (arrow). The same lesion is shown on axial FLAIR **(B)** and axial DIR **(C)** images. ADC **(D)** and DWI **(E)** show no evidence of restricted diffusion. Created in BioRender. Puthenparampil, (M) (2026) https://BioRender.com/t14zfgo.

Since the Expanded Disability Status Scale was 3.0 (best visual acuity in the affected eye: 10/200, Visual Functional System Score 5), treatment with *ublituximab* was started. After one year of follow-up no evidence of clinical or radiological disease activity has been observed.

## 3 Discussion

OPN is a rare inflammatory disorder in which the inflammation is confined to optic nerve sheath. First described by Edmunds and Lawford, OPN usually presents with pain, disc oedema and various features of optic nerve dysfunction. Indeed, clinically it often mimics acute ON, thus resulting in late diagnosis and treatment. OPN can be idiopathic or secondary to underlying systemic autoimmune disorder, such as Antineutrophil Cytoplasmic antibodies (ANCA)-associated vasculitis, Systemic Lupus Erythematosus (SLE), sarcoidosis, syphilis and Crohn’s disease (8). However, its involvement in MOGAD has largely been described, as well as also in a few patients with MS (3%) (4).

Although histological findings have never been described, MRI shows an inflammation restricted to the dura mater and the arachnoid. Chronic inflammation, mediated by T and B lymphocytes, eosinophils, and macrophages, activates fibroblasts to induce collagen deposition, and ultimately leads to tissue proliferation and dural thickening (9). The optic nerve (and its surrounding pia mater) is in tight proximity but not in continuity, so that inflammation does not directly involve the optic nerve. Indeed, the diagnosis of OPN is driven by MRI findings (especially fat-suppressed, Gadolinium-enhanced T1 weighted MRI of the orbits). Unlike ON, wherein optic nerve enhancement is seen on MRI, the characteristic finding in OPN is the contrast enhancement of the optic nerve sheath with sparing of the optic nerve, appearing as ‘Tram Tracks’ in the axial view and as a ‘Doughnut’ in the coronal view (10). Optic nerve sheath meningioma can be differentiated on the basis of calcifications, which can be seen using CT if the diagnosis is unclear (11). It is important to not confuse the tram track sign with normal enhancement of the dural sheath due to its rich vascular supply (12).

From an immunopathogenic point of view, it is not surprising that OPN might be also associated with MOGAD (frequently) and NMOSD (more rarely), since in these disorders the adaptive immune system is licensed to target CNS in peripheral lymphoid tissues. Indeed, the mechanisms behind the lack of tolerance towards CNS-restricted self-antigens, might also increase the risk to the development of additional self-targets. This has already been observed in NMOSD, where an association with systemic autoimmune disorders (SLE; Sjögren syndrome, SS) has been already described (13, 14). Therefore, the immunological screening is pivotal in the presence of OPN, but it is also important to exclude MOGAD and NMOSD testing for anti-AQP4 and -MOG antibodies.

The detection of antiphospholipid antibodies might be particularly challenging. As occurred in our case (positivity of anti-β_2_glycoprotein1 and anti-cardiolipin IgM antibodies), the ischemic etiology (and thus APS) needs to be evaluated by fluorescein angiography (for optic nerve damage), or by pathological diffusion weighted signal (ADC and DWI, for CNS lesion). In addition, also the association with SLE needs to be carefully excluded (15). Even in absence of APS, optic neuritis and other MS-like neurological manifestations have been reported in antiphospholipid antibody (aPL)-positive patients (15). Therefore, the possibility of an aPL-mediated vascular or immune-mediated mechanism was carefully considered in the differential diagnosis of our patient. However, despite persistent aPL positivity, the patient had no history of thrombotic or obstetric events and did not fulfil any of the six clinical domains included in the 2023 ACR/EULAR classification criteria for APS (16).

Since in our case retinal fluorescein angiography was negative and the temporal white matter lesion did not demonstrate any diffusion restriction on DWI/ADC sequences, the ischemic etiology (and thus APS) was ruled out. Unfortunately, susceptibility-based MRI biomarkers of MS, including the central vein sign and paramagnetic rim lesions, could not be reliably assessed on the available MRI sequences, representing a limitation of the present case.

In presence of OPN with or without ON, OCT findings might further contribute to the diagnostic process. Indeed, it is widely known that in the very early stage of ON, OCT may be normal, especially in MS, where observable atrophic changes, involving primarily the temporal sector of peripapillary retinal nerve fiber layer (RNFL) and the papillary-macular bundle, often require at least three months to reach a size detectable by standard OCT measurements (17). On the other hand, partial exception is made for MOGAD where OCT can be considered as a relevant acute-stage diagnostic tool in patients with ON, since in MOGAD a significantly greater peripapillary pRNFL thickening is demonstrated (118 μm cutoff, 74% sensitivity and 82% specificity) compared to MS, providing a valuable biomarker for differentiating these conditions early (18). Although in our case the absence of pRNFL thickening and asymmetry was not supportive for MOGAD, the correct timing (before any treatment) and methodology (tested by l-CBA) were crucial to exclude MOGAD definitively (3).

At the time of the patient’s presentation in February 2024, the 2017 McDonald criteria represented the applicable diagnostic framework (7). Therefore, the initial diagnostic approach was not related to unfamiliarity with new criteria (that were not yet published) (1). In retrospect, the revised criteria would have permitted an MS diagnosis at presentation, once alternative explanations had been carefully excluded (7). Nevertheless, the appearance of a new right temporal juxtacortical lesion after 12 months, together with the pre-existing temporal periventricular lesion, MRI-confirmed optic nerve involvement, and CSF-restricted IgG oligoclonal bands, ultimately supported the diagnosis of MS. The immunological basis of an OPN phenotype in MOG-IgG-negative MS remains unknown. One intriguing possibility is antigen-specific cellular autoimmunity. We previously identified CSF-infiltrating MOG_35-55_-reactive CD4+ T cells in anti-MOG antibody-negative patients with MS and showed that different T-cell specificities may contribute to MS heterogeneity (19). T-cell reactivity against MOG or other CNS targets could therefore be investigated in future patients with similar atypical phenotypes, although this hypothesis could not be examined in the present case.

Interestingly, according to the most recent MS diagnostic criteria, with the inclusion of optic nerve as the fifth anatomical location to establish DIS, the diagnosis might have been established earlier (1). This observation highlights the diagnostic risk of over-interpreting early immunological and radiological red flags in optic neuritis. Although such findings may initially suggest MOGAD or systemic autoimmune disorders, premature diagnostic labelling may obscure the possibility of an evolving multiple sclerosis phenotype, particularly in seronegative patients. However, in this particular case, the atypical clinical presentation and the persistent high-titer aPL positivity required further exclusion of systemic and CNS-restricted autoimmune disorders in accordance with current recommendations (1).

Finally, it is of interest to evaluate the sensitivity and specificity of MRI, OCT, and VEPs in acute and chronic phases of optic neuritis (17, 20). Indeed, in our case VEPs were abnormal from symptom onset, while MRI identified the optic nerve lesion one month after the onset. Clarifying the timing of diagnostic tests used to support optic nerve involvement is relevant for the diagnosis of both ON and MS.

In conclusion, this case emphasizes that MRI and OCT may provide early clues and red flags to differentiate MS from NMOSD, MOGAD or other autoimmune systemic diseases; however, these findings should be interpreted cautiously within a longitudinal and integrative diagnostic framework. Recognition of this dynamic diagnostic process is essential to avoid premature diagnostic labelling and to ensure timely initiation of appropriate disease-modifying therapy.

## 4 Patient perspective

The patient reported that the sudden onset of visual loss was alarming, particularly because it occurred without pain and progressed rapidly. She described the diagnostic pathway as stressful, as multiple possible conditions were considered and definitive answers were not immediately available. The uncertainty during follow-up, while different diagnostic hypotheses were being explored, represented a challenging aspect of her clinical experience. Over time, the progressive clarification of the diagnosis helped her better understand the rationale behind repeated investigations and therapeutic choices. Although visual recovery remained only partial, she reported that regular follow-up and clear communication with the clinical team contributed to a greater sense of involvement and reassurance.

The patient emphasized that understanding the evolving nature of the diagnostic process helped her adapt to long-term monitoring and treatment decisions. Since no adverse event has been reported and no evidence of disease activity has been described, the patient felt reassured and motivated to continue the treatment.

## Data Availability

The original contributions presented in the study are included in the article/supplementary material. Further inquiries can be directed to the corresponding author.
